# Exploring the association among the tryptophan to serotonin and kynurenine pathways, cognition and suicidal behaviour: a secondary analysis in a sample of individuals affected by Bipolar Disorder.

**DOI:** 10.1192/j.eurpsy.2023.265

**Published:** 2023-07-19

**Authors:** P. Paribello, M. Manchia, A. Squassina, C. Pisanu, D. Congiu, S. Dall’Acqua, S. Sut, S. Nasini, M. Garzilli, B. Guiso, F. Suprani, V. Pulcinelli, M. N. Iaselli, I. Pinna, G. Somaini, L. Arru, C. Corrias, F. Pinna, S. Comai, B. Carpiniello

**Affiliations:** 1 Department of Medical Sciences and Public Health, Unit of Clinical Psychiatry, University Hospital Agency of Cagliari, Cagliari, Italy; 2 Department of Pharmacology, Dalhousie University, Halifax, Canada; 3 Department of Biomedical Science, Section of Neuroscience and Clinical Pharmacology, University of Cagliari, Cagliari; 4 Department of Pharmaceutical and Pharmacological Sciences; 5 Department of Biomedical Sciences, University of Padova, Padova; 6 San Raffaele Scientific Institute, Milan, Italy; 7 Department of Psychiatry, McGill University, Montreal, Canada

## Abstract

**Introduction:**

Stroop test iteration performances and metabolism of tryptophan (TRP) via serotonin (5-HT) and kynurenine (KYN) have both been associated with suicidal behaviors. This study aims to probe their possible interactions.

**Objectives:**

We explored the association of the performances on the Emotion Inhibition Subtask (EIS) of the Brief Assessment of Cognition for Affective Disorder and the plasmatic levels of 5-hydroxytryptophan (5-HTP), 5-HT, KYN, melatonin (MLT) among subjects with Lifetime Suicidal Ideation (LSI) vs non-LSI, and with Lifetime Suicide Attempts (LSA) vs non-LSA.

**Methods:**

Using R studio, we employed: 1) the t-test for parametric data and the Wilcoxon test for non-parametric data; 2) Linear Modeling to probe the associations of EIS performances with MLT, KYN, 5-HTP or 5-HT plasmatic levels.

**Results:**

In a sample comprising 45 individuals affected by Bipolar Disorder, we found a statistically significant difference for the Color Naming (CN, image 1) and the Neutral words (NW) subtasks among LSA vs non-LSA. In LSI vs non-LSI, only the NW retained significance, but not the CN. A significant association emerged between CN and 5-HTP in LSI but not in non-LSI (image 2). Similarly, in LSA, an association was found between CN and 5-HTP, but not in non-LSA (image 3). No statistically significant difference emerged among groups regarding gender composition, age, pharmacological therapy, Body Mass Index, Hamilton Depression Rating Scale, Young Mania Rating Scale, or Clinical Global Impression scale - Severity.

**Image:**

**Figure:**
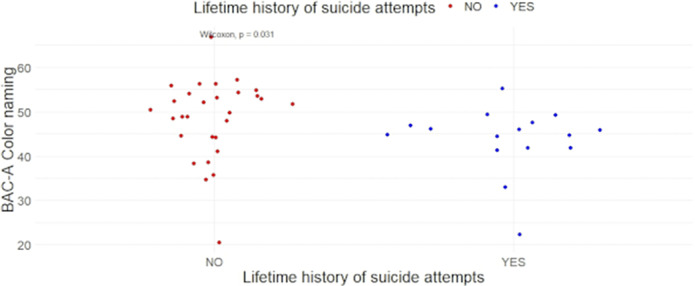

**Image 2:**

**Figure:**
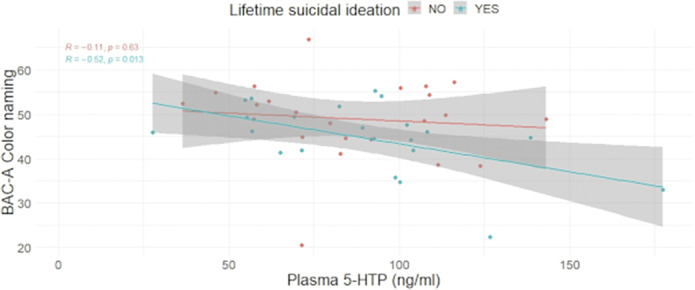

**Image 3:**

**Figure:**
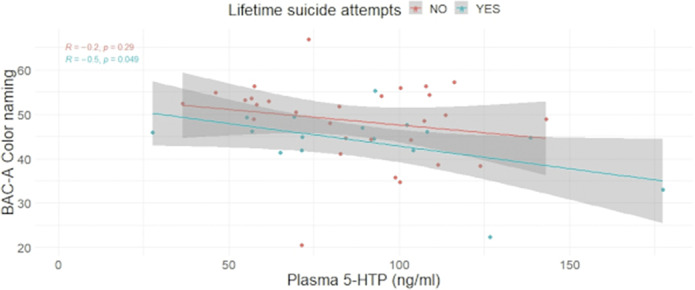

**Conclusions:**

We found that the plasmatic levels of the metabolites of TRP via 5-HT were correlated to some EIS performances. These findings may represent a hypothesis-generating platform for further investigations.

**Disclosure of Interest:**

None Declared

